# Elevated CO_2_ Drives Cadmium Phytostabilization in the *Robinia pseudoacacia*–Rhizobia Symbiosis by Altering Cadmium Bioavailability, Nutrient Uptake and Antioxidant Systems

**DOI:** 10.3390/toxics14090752

**Published:** 2026-08-26

**Authors:** Xun Wang, Ruoshi Wang, Shaoxiong Lin, Ming Ma, Sixi Zhu

**Affiliations:** 1 The Karst Environmental Geological Hazard Prevention of Key Laboratory of State Ethnic Affairs Commission, College of Eco-Environment Engineering, Guizhou Minzu University, Guiyang 550025, China; wangxun116@126.com (X.W.);; 2 School of Economics and Management, Guizhou Normal University, Guiyang 550025, China; 3 Center of Molecular Ecophysiology (CMEP), College of Resources and Environment, Southwest University, Chongqing 400715, China

**Keywords:** elevated CO_2_, cadmium, phytostabilization, *Robinia pseudoacacia*, symbiosis

## Abstract

Elevated atmospheric carbon dioxide (ECO_2_) is a key climatic factor influencing the resilience of plant–microbial symbiotic systems against heavy metal contamination. *Robinia pseudoacacia*–rhizobia symbiosis shows great potential for cadmium (Cd) remediation. However, the mechanism by which ECO_2_ regulates Cd phytostabilization in symbiosis remains unclear. This study conducted a 90-day experiment in growth chambers to investigate the effects of ECO_2_ on the growth, Cd accumulation and chemical forms, as well as nutrient uptake and antioxidant system in *Robinia pseudoacacia*–rhizobia symbiosis. Results indicated that ECO_2_ significantly increased plant biomass and photosynthetic efficiency while significantly raising Cd content in roots (34.5%, *p* < 0.001) and decreasing it in shoots (31.4%, *p* < 0.001). This resulted in a significant reduction in Cd translocation factor (TF). Meanwhile, ECO_2_ markedly increased Cd accumulation in roots (81.2%, *p* < 0.001) and reduced the bioavailability of Cd in the symbiosis. Moreover, ECO_2_ promoted the content of nutrients and stimulated the antioxidant system. The random forest model indicated that root weight, Cd and Mn contents are the core factors for ECO_2_-driven Cd phytostabilization. This study demonstrates that ECO_2_ enhanced Cd phytostabilization by optimizing the resistance of symbiosis to Cd, offering a novel perspective for predicting plant–microbe joint restoration of heavy metal pollution under global climate change scenarios.

## 1. Introduction

Rising atmospheric CO_2_ concentration is a key indicator of global climate change and has steadily increased from 280 ppm to the current level of 415 ppm [1]. CO_2_ concentrations are expected to reach 650 ppm by the middle of this century, which will have a profound impact on material cycles and biological interactions in global ecosystems [2]. Previous studies reported that elevated CO_2_ (ECO_2_) could stimulate photosynthesis in plants, thereby promoting plant growth and increasing crop yields [3,4]. In addition, ECO_2_ serves a critical role in plant defense against abiotic stresses, including drought, salinity, and heavy metals [5].

Cadmium (Cd) pollution has become a persistent environmental problem in farmlands worldwide due to increasing human activities such as industrial smelting and agricultural non-point source inputs [6]. Because of its high mobility and strong biological toxicity, Cd can accumulate through the soil–plant system, not only inhibiting plant growth and metabolism but also posing a threat to human health through the food chain [7]. Consequently, the remediation of soil Cd contamination has become a key research priority in the field of ecological and environmental conservation [8].

Phytostabilization employs root systems and the rhizosphere microenvironment to immobilize and passivate heavy metals, thereby reducing their bioavailability [9]. This is a key approach to remediating soil contamination caused by heavy metals. Black locust (*Robinia pseudoacacia* L.) is an optimal tree species for the remediation of Cd-contaminated soil owing to its strong stress tolerance, well-developed root system, and outstanding nitrogen-fixing ability [10]. Nitric oxide upregulated genes involved in root cell wall biosynthesis, facilitating cell wall thickening under Cd exposure, thereby enhancing the Cd tolerance of *Robinia pseudoacacia* [11]. The symbiotic relationship between black locust and rhizobia can enhance adaptation to Cd-contaminated environments by regulating the rhizosphere microenvironment and improving nutrient uptake in plants [12,13]. The combined inoculation of rhizobia and AMF promoted the growth of black locust and enhanced its resistance to Cd [14]. The synergistic interaction between beneficial rhizosphere bacteria and rhizobia improved the Cd phytoremediation by *Robinia pseudoacacia* in lead–zinc mining areas [15]. After 5 years of vegetation restoration, black locust reduced the bioavailability of Cd in zinc tailings, indicating that black locust is a promising candidate tree for Cd remediation [16]. Moreover, a previous study revealed that ECO_2_ (750 ppm) enhanced the resistance of *Robinia pseudoacacia* to Cd by promoting the synthesis of total flavonoids [17]. Moreover, ECO_2_ (700 ppm) promoted Cd stabilization in the rhizosphere soil of black locust by facilitating glomalin accumulation [18]. Another study indicated that ECO_2_ contributed to Cd sequestration by promoting glomalin-related soil protein accumulation in the rhizosphere of black locust [19]. ECO_2_ raised SOD activity while reducing the diversity of arbuscular mycorrhizal fungi (AMF) community in the root of *Robinia pseudoacacia* [20]. Glomus mosseae lowered Cd levels and flavonoid synthesis in *Robinia pseudoacacia* exposed to ECO_2_ (750 ppm) [21]. It is evident from the above that, in the context of elevated CO_2_ levels, black locust has considerable practical value for the remediation of Cd-contaminated soil.

However, the current understanding of the mechanism by which ECO_2_ enhances Cd resistance of *Robinia pseudoacacia*–rhizobia symbiosis remains unclear. This study examined the regulatory effects of ECO_2_ on Cd phytostabilization in symbiosis by analyzing Cd accumulation, bioavailability, nutrient content, and antioxidant systems. We proposed three hypotheses: (1) ECO_2_ stimulates photosynthesis and promotes the growth of *Robinia pseudoacacia*–rhizobia symbiosis; (2) ECO_2_ alters Cd accumulation, bioavailability, and nutrient content; (3) ECO_2_ activates the antioxidant system and relieves Cd-induced oxidative stress.

## 2. Materials and Methods

### 2.1. Plant Cultivation and Growth Conditions

This experiment was conducted at the National Purple Soil Fertility Monitoring Station at Southwest University in Beibei, Chongqing. The seeds of *Robinia pseudoacacia* L. were obtained from a commercial seed store in Hebei Province, China. Uniformly plump seeds were soaked in concentrated sulfuric acid for 10 min, then in 95% alcohol for 2 min. Afterward, the seeds were sterilized with sodium hypochlorite for 10 min and finally rinsed five times with sterile water. The seeds were germinated in a climate chamber at 20 °C and 300 μmol m^−2^ s^−1^ photosynthetic active radiation for 16 h per day. Once the seedlings of black locust had grown to about 5 cm, they were transplanted into plastic pots in the greenhouse. Each plastic pot is 20 cm height and 15 cm in diameter and contains 2.5 kg of sterilized substrate (90% sand and 10% vermiculite). The seedlings were watered every 2 days and apply 200 mL full nutrient solution every 4 days [12]. The nutrient solution formula is as follows: 1 mM KNO_3_, 1 mM NH_4_NO_3_, 0.05 mM K_2_SO_4_, 0.25 mM MgSO_4_·7H_2_O, 1 mM CaCl_2_, 0.5 mM KH_2_PO_4_, 0.01 mM Ferric citrate, 2 μM H_3_BO_3_, 1 μM MnSO_4_⋅H_2_O, 0.2 μM CuSO_4_⋅5H_2_O, 0.1 μM CoSO_4_⋅7H_2_O, 0.5 μM ZnSO_4_·7H_2_O, 0.1 μM Na_2_MoO_4_⋅2H_2_O.

The seedlings were transferred to the CO_2_ enrichment growth chambers after growing in the greenhouse for a month. The experimental facility comprises 12 rectangular growth chambers, each measuring 1 m wide, 1.5 m long, and 2.5 m in height [22]. Each rectangular growth chamber contained 40 pots of black locust seedlings. The CO_2_ concentrations were set at 415 ppm (ambient CO_2_, control), 600 ppm (Low Elevated CO_2_, LECO_2_), 800 ppm (Elevated CO_2_, ECO_2_) and 1000 ppm (High Elevated CO_2_, HECO_2_). Each treatment involved 4 biological replicates. During CO_2_ treatment, rhizobia inoculation of black locust seedlings was conducted using strain *Mesorhizobium huakuii* QD9. This strain of rhizobia was isolated from black locust forest in Qingdao, Shandong, China, and preserved in the Centre of Molecular Ecophysiology at Southwest University. A previous study has described the cultivation process of *Mesorhizobium huakuii* QD9 strain in detail [23]. The strain was removed from the −80 °C ultra-low temperature freezer, streaked onto Yeast Mannitol Agar (YMA) solid medium, and incubated at 28 °C for activation. After single colonies appeared on the medium, a single colony was picked with a sterile bamboo stick, inoculated into Tryptone Yeast (TY) liquid medium, and incubated at 28 °C with shaking at 150 rpm for 2 days. The cultured bacterial suspension was transferred into a 50 mL sterile centrifuge tube and centrifuged at 5000 rpm for 10 min. The supernatant was discarded, and the rhizobia pellet was resuspended in sterile normal saline to adjust the OD_600_ to 0.8–1.0 A total of 10 mL of rhizobia suspension was obtained using a sterile syringe and inoculated onto the roots of black locust. Inoculation was conducted every 5 days until obvious root nodules formed.

### 2.2. Experimental Design

**Figure 1:**
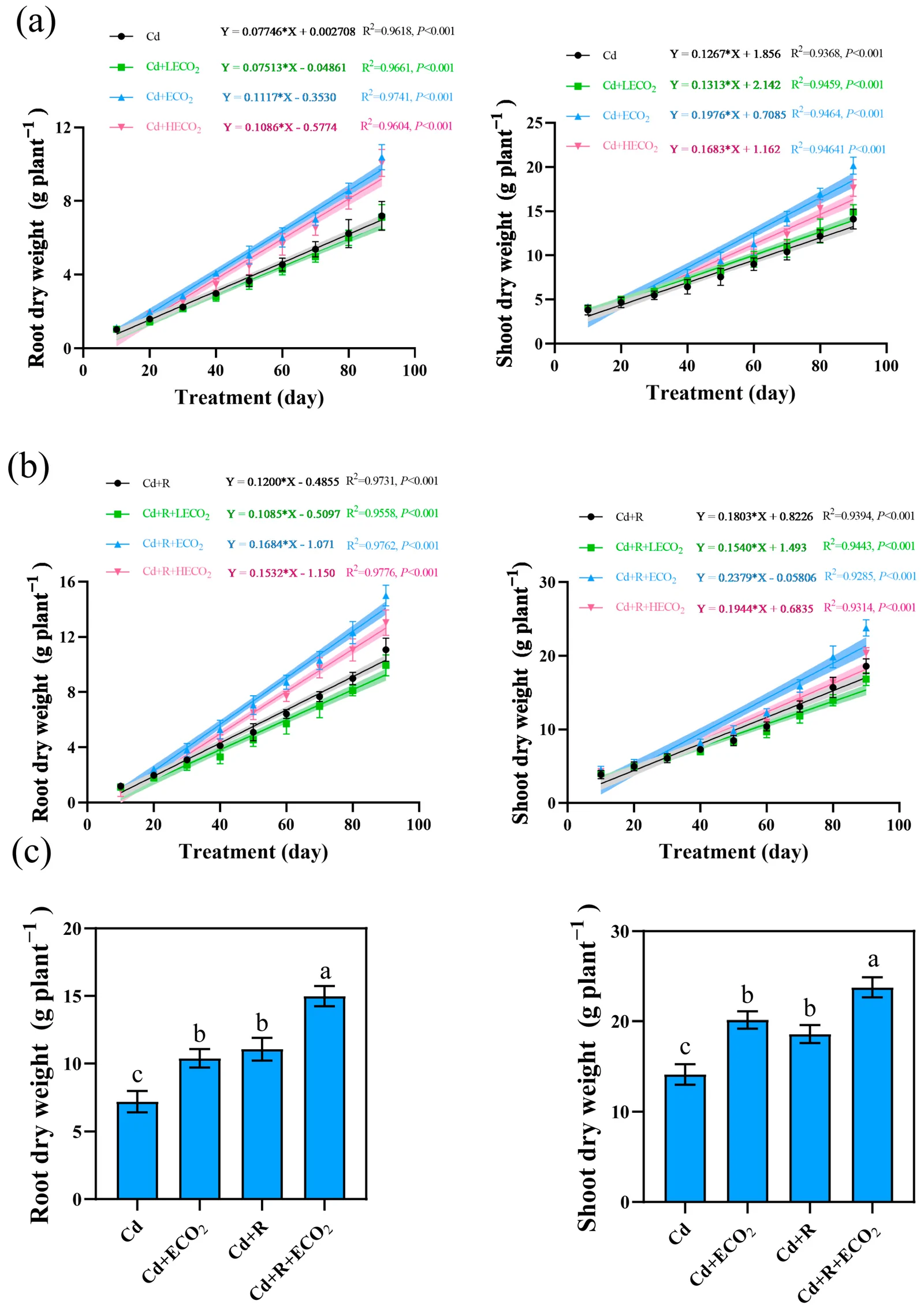
Effects of CO_2_ on the growth of *Robinia pseudoacacia*–rhizobia symbiosis under Cd exposure. (**a**) Linear regression of different CO_2_ concentrations on the growth of *Robinia* over a 90-day treatment period. (**b**) Linear regression of different CO_2_ concentrations on the growth of *Robinia pseudoacacia*–rhizobia symbiosis over a 90-day period. (**c**) Dry weight of the roots and shoots of *Robinia pseudoacacia*–rhizobia symbiosis after 90 days of treatment. Different lowercase letters denote significant differences among treatments at the *p* < 0.05 level. The colored areas represent the 95% confidence interval.

After three months of cultivation in the CO_2_ enrichment growth chambers, Cd treatments were conducted with concentration set at 10 mg kg^−1^. According to the risk control standard for soil contamination of Chinese agricultural land (GB15618-2018 [24]), which was issued by the Ministry of Ecology and Environment of China, the risk control values for Cd are 1.5–4.0 mg kg^−1^ [25]. In addition, the average concentration of Cd in industrial areas soils of China is 23.7 mg kg^−1^ [26]. Therefore, this study used an intermediate value of 10 mg kg^−1^ as the Cd treatment concentration. A total of 200 mL of cadmium chloride (CdCl_2_) solution was added to each culture substrate (25 mg pot^−1^) to reach a Cd concentration of 10 mg kg^−1^. In order to determine the optimal CO_2_ treatment concentration, this study conducted a 90-day preliminary experiment with three different CO_2_ concentrations: 600 ppm (LECO_2_), 800 ppm (ECO_2_), and 1000 ppm (HECO_2_). The results showed that in the non-inoculated rhizobia group, ECO_2_ and HECO_2_ treatments promoted the growth of black locust roots and shoots, while the LECO_2_ treatment did not exhibit a significant promoting effect. Among these, the ECO_2_ treatment demonstrated a greater increase in growth throughout the 90-day treatment period. Similarly, in the rhizobia inoculation group, ECO_2_ and HECO_2_ treatments stimulated the growth of both the roots and shoots of black locust, but the LECO_2_ treatment inhibited black locust growth (Figure 1a,b). In addition, a previous study simulated that CO_2_ concentrations may exceed 800 ppm by the end of the 21st century [27]. Therefore, this study selected 800 ppm (ECO_2_) as the CO_2_ treatment concentration. This study set up four treatments: (1) Cd (10 mg kg^−1^); (2) Cd+ECO_2_ (10 mg kg^−1^ Cd + 800 ppm CO_2_); (3) Cd+R (10 mg kg^−1^ Cd+rhizobia inoculation); (4) Cd+R+ECO_2_ (10 mg kg^−1^ Cd+rhizobia inoculation + 800 ppm CO_2_). Four biological replicates were set up for each treatment. The *Robinia*–rhizobia symbiosis was harvested following a period of 90 days of experimental treatment in the CO_2_ enrichment growth chambers. Fresh tissues were collected to measure physiological and biochemical parameters.

### 2.3. Determination of Plant Growth and Photosynthetic Parameters

The fresh roots and shoots were cut into small pieces and then dried in an oven at 105 °C for 5 h, and then placed at 75 °C until constant weight was reached. The photosynthetic parameters of black locust leaves were measured by Li-6800 portable photosynthesis system (Li-cor, Lincoln, NE, USA). The measurement was carried out on a sunny day between 8:00 a.m. and noon. Four healthy leaves were selected from each black locust to determine foliar photosynthetic parameters. The chlorophyll a, chlorophyll b, total chlorophyll, and carotenoids content were determined using an assay kit, following the experimental procedures provided by the manufacturer (Grace Biotechnology, Suzhou, China) [11]. A total of 0.1 g of black locust leaves was cut into small pieces, and placed them in a mortar. Then 1 mL of 95% ethanol, a small amount of quartz sand, and calcium carbonate powder were added. This mixture was ground in the dark until a homogeneous paste was formed; this homogenate was then transferred to a 10 mL test tube and made up to 10 mL with 95% ethanol. This was extracted in the dark for 3 h, until the tissue residue turned completely white. A total of 200 μL of the extract and 200 μL of 95% ethanol were added, and the resulting mixture was transferred to a 96-well plate; a multi-mode microplate reader (TriStar2, LB942, Berthold, Germany) was used to measure the absorbance at wavelengths of 649 nm, 665 nm, and 470 nm, respectively.

### 2.4. Determination of Cd Accumulation, Chemical Form Fractions and Nutrients Content

Plant tissues were immersed in a solution of 10 mM ethylenediaminetetraacetic acid disodium salt (EDTA-2Na) for 10 min, followed by drying in an oven at 75 °C for 48 h until constant weight was achieved. A 0.2 g powder sample was digested in a mixture of HNO_3_ and H_2_O_2_ (3:1, *v*/*v*) in a conical flask at 200 °C on an electric heating plate for 3 h. Once the digestion was complete, the solution was filtered and titrated with ultrapure water to 10 mL. The Cd and nutrients contents were measured using an inductively coupled plasma optical emission spectrometer (ICP-OES) (Agilent 5110, Agilent Technologies Inc., Santa Clara, CA, USA) [28]. The accuracy of the Cd and nutrients contents were calibrated using the certified reference material (GBW07603) from the Centre for Standard Reference of China [12]. The translocation factor (TF) is calculated by dividing the shoot Cd content by the root Cd content [29]. The Cd accumulation = Cd content in tissue x dry weight of tissue [30]. The chemical forms of Cd fractions were determined as previously described [31]. A 0.5 g sample of fresh roots and 0.5 g of fresh shoots were weighed separately, 20 mL of extraction solution was added, and these were ground to a homogeneous mixture. After shaking at 25 °C for 24 h, the mixture was centrifuged at 5000× *g* for 10 min and the supernatant was collected. Another 10 mL of extraction solution was added to the residue, shook for 2 h, and then centrifuged at 5000× *g* for 10 min. The supernatant was then collected. The supernatant is used to determine the Cd content in the corresponding form, while the residue is used for the next step of chemical form extraction. The sequence for adding extraction solutions is as follows: (1) 80% ethanol, to extract inorganic Cd; (2) deionized water, to extract water-soluble Cd; (3) 1 M NaCl, to extract protein-bound Cd and pectate; (4) 2% acetic acid (HAc) to extract phosphate Cd, which is poorly soluble in water; (5) 0.6 M hydrochloric acid (HCl) to extract oxalates- Cd; (6) the remaining Cd exists in the form of residue.

### 2.5. Determination of Antioxidant System Parameters

The malondialdehyde (MDA) content was measured using the thiobarbituric acid method, as previously described [32]. The absorbance of the extract was measured at 532 nm and 600 nm using a multi-mode microplate reader (TriStar2, LB942, Berthold, Germany). The superoxide anion (O_2_^−^) content was quantified at 540 nm absorbance using an improved assay. The hydrogen peroxide (H_2_O_2_) content was assessed at 415 nm using a modified titanium sulfate method [33]. Superoxide dismutase (SOD) activities were determined using the nitroblue tetrazolium (NBT) method. We weighed 0.1 g of fresh root and added it to 1 mL of extract for ice bath homogenization. The mixture was then centrifuged at 12,000 rpm for 10 min at 4 °C. The supernatant was collected and the reaction solution was added sequentially. The absorbance of the extract was measured at 450 nm using a multi-mode microplate reader [34]. The catalase (CAT) activities were determined using the assay kits provided by Suzhou Grace Biotechnology. The absorbances of the extracts were determined at 510 nm using a multi-mode microplate reader [14]. Peroxidase (POD) and glutathione reductase (GR) activities were measured using the assay kits, following the experimental protocols provided by the manufacturer (Grace Biotechnology, Suzhou, China). The absorbances were measured at 470 nm and 412 nm, respectively [13]. The levels of glutathione (GSH) and ascorbic acid (AsA) were measured using the assay kits (Grace Biotechnology, Suzhou, China). The absorbances were measured at 412 nm and 534 nm, respectively.

### 2.6. Statistical Analysis

The raw data were initially analyzed for normal distribution using the Shapiro–Wilk test and for homogeneity of variances using the Levene test in SPSS Statistics 23 (version R23.0.0) (IBM, Armonk, NY, USA). Logarithmic transformation was applied if the data were not normally distributed. The statistical analyses were conducted using one-way ANOVA. The Least Significant Difference and Duncan’s tests were applied at a significant level of *p* < 0.05 using SPSS 23. The results were shown as the mean ± standard deviation (SD) (n = 4, four biological replicates). The figures were produced using GraphPad Prism 9 and R Studio software R 4.3.1.

The results of the random forest model were visualized using R Studio software. Random forest modeling was performed in the R software (R 4.3.1.) environment using the tidymodels framework, with the random forest package specified as the computational engine. The data set was partitioned using the createDataPartition function in the caret package. Data preprocessing, hyperparameter optimization, and model performance evaluation were conducted using the recipes, tune, and yardstick components, respectively. In addition, the iml (version 0.11.4), fastshap (version 0.1.1), and shapviz (version 0.9.7) packages were used to evaluate variable importance and interpret the model using SHAP values. Model performance was evaluated using the root mean square error (RMSE), coefficient of determination (R^2^), and mean absolute error (MAE). The parameter combination with the lowest cross-validated RMSE was selected as the optimal combination. The final model was then refitted to the complete training data set using the optimized parameters, and its generalization performance was evaluated using the independent testing data set.

## 3. Results

### 3.1. Selection of CO_2_ Concentrations Treatment and Plant Growth

The Cd+ECO_2_ treatment significantly increased the dry weight in roots and shoots by 44.3% and 42.8% compared to Cd treatment alone (Figure 1c). The Cd+R+ECO_2_ treatment also markedly promoted the growth of both the roots (35.4%, *p* < 0.01) and shoots (27.9%, *p* < 0.01) of *Robinia*–rhizobia symbiosis, compared to Cd+R treatment (Figure 1c). The results indicated that ECO_2_ promoted the growth of *Robinia*–rhizobia symbiosis under Cd exposure.

### 3.2. Effects of ECO_2_ on CO_2_ and H_2_O Gas Exchange and Leaf Pigments

**Figure 2:**
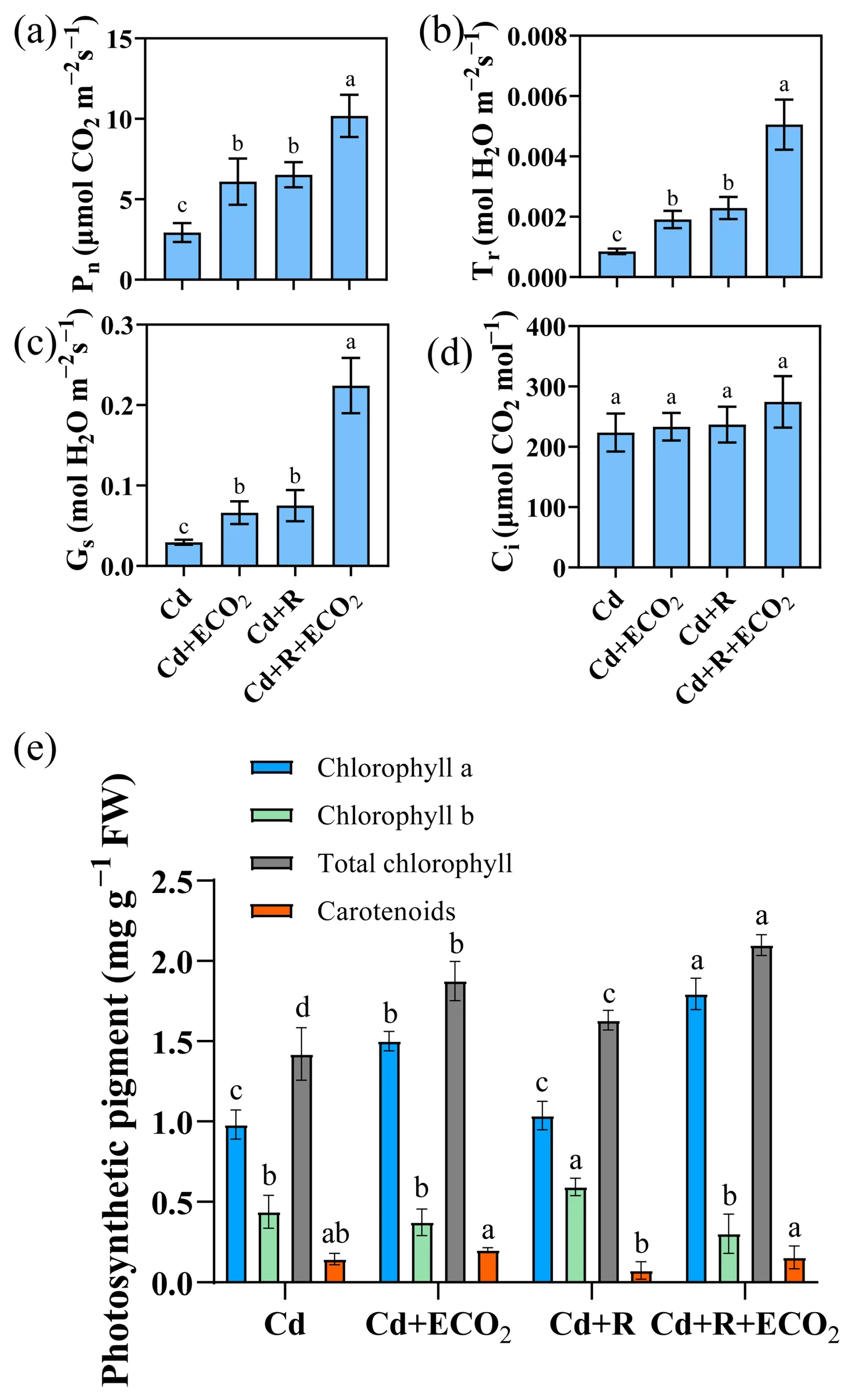
Effects of ECO_2_ on photosynthetic parameters. (**a**) Pn: net rate of photosynthesis, (**b**) Tr: transpiration, (**c**) Gs: stomatal conductance, (**d**) Ci: intercellular CO_2_ concentration, (**e**) photosynthetic pigments in leaves. Different lowercase letters denote significant differences among treatments at the *p* < 0.05 level.

The Cd+R+ECO_2_ treatment significantly improved Pn, Tr, and Gs, with no obvious effect on Ci compared to Cd+R treatment (Figure 2a–d). Likewise, the Cd+R+ECO_2_ treatment greatly raised the content of photosynthetic pigments in black locust leaves (Figure 2e). The above results indicated that ECO_2_ enhanced photosynthesis in *Robinia*–rhizobia symbiosis leaves.

### 3.3. Effects of ECO_2_ on Cd Content

**Figure 3:**
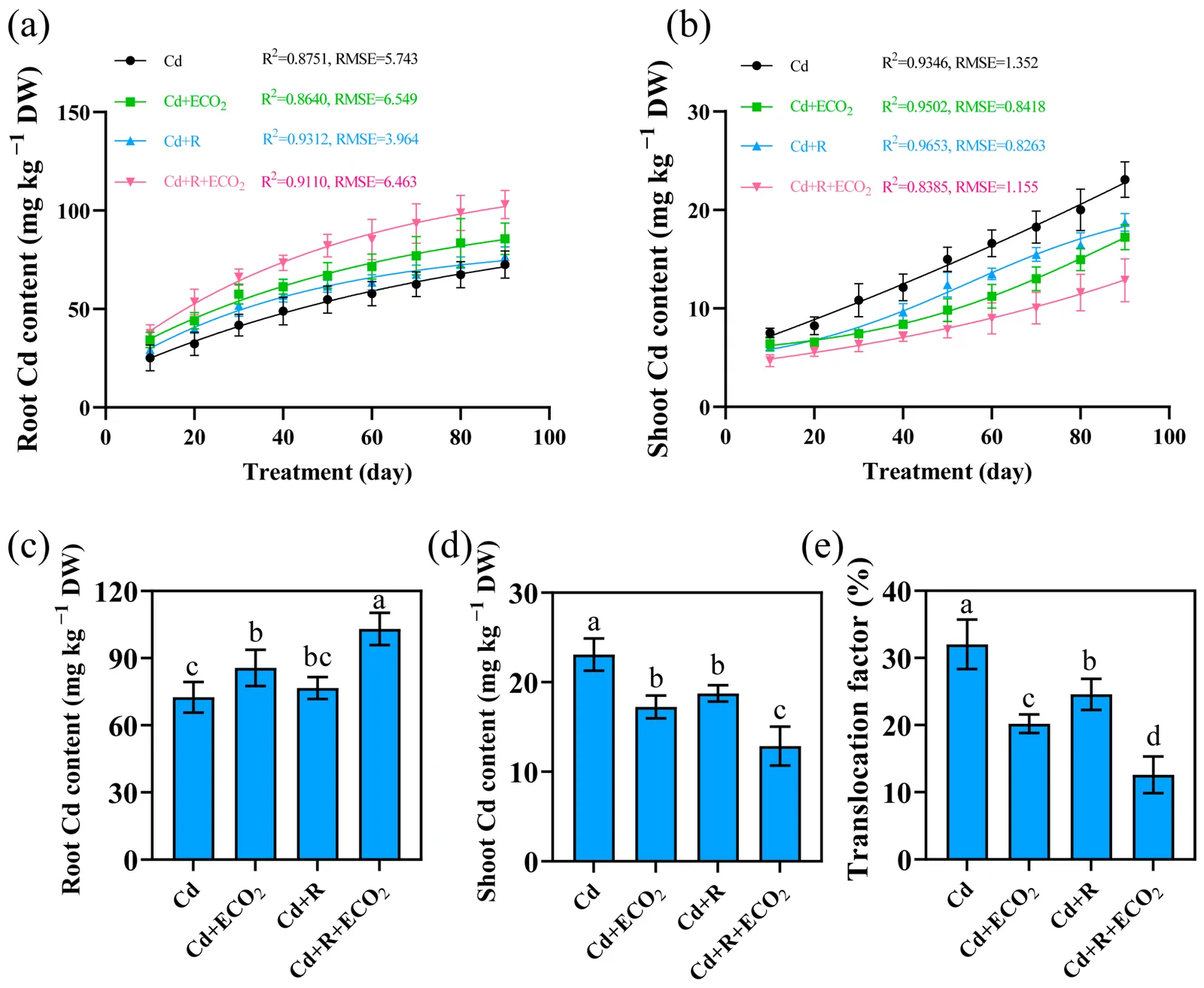
Effects of ECO_2_ on Cd content. (**a**) Nonlinear fit of ECO_2_ on root Cd content over a 90-day treatment period. (**b**) Nonlinear fit of ECO_2_ on shoot Cd content over a 90-day treatment period. (**c**) Root Cd content after 90-day treatment. (**d**) Shoot Cd content after 90 days treatment. (**e**) Cd translocation factor. Different lowercase letters denote significant differences among treatments at the *p* < 0.05 level.

During the 90-day treatment period, the Cd+ECO_2_ treatment increased Cd content compared to Cd treatment. Meanwhile, the Cd+R+ECO_2_ treatment further raised Cd content in roots compared to Cd+R treatment (Figure 3a). In contrast, the Cd content in Cd+ECO_2_ treatment consistently remained lower than that in Cd treatment within the black locust shoots. Moreover, during the 90-day treatment period, the Cd+R+ECO_2_ treatment exhibited the lowest Cd content in shoots (Figure 3b). The Cd+R+ECO_2_ treatment significantly elevated Cd content in roots (34.5%, *p* < 0.001) while decreasing Cd content in shoots (31.4%, *p* < 0.001), resulting in a significant 48.8% reduction in TF compared to Cd+R treatment (Figure 3c–e). The results showed that ECO_2_ increased Cd content in roots while decreasing Cd content in shoots, thereby inhibiting Cd translocation from roots to shoots.

### 3.4. Effects of ECO_2_ on Cd Accumulation in Robinia–Rhizobia Symbiosis

**Figure 4:**
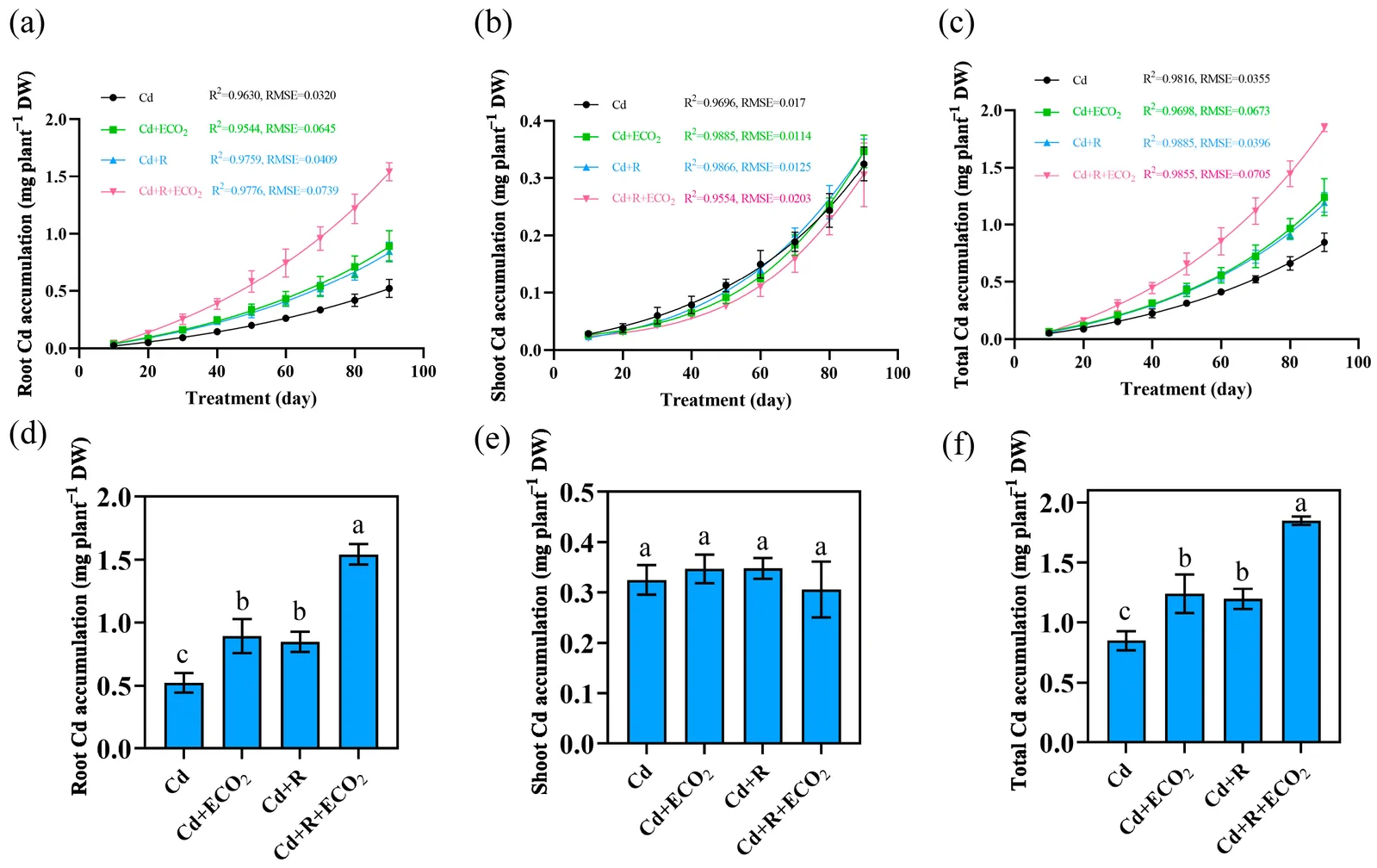
Effects of ECO_2_ on Cd accumulation in *Robinia*–rhizobia symbiosis. (**a**) Nonlinear fit of ECO_2_ on root (**a**) and shoot (**b**) Cd accumulation over a 90-day treatment period. (**c**) Nonlinear fit of ECO_2_ on total Cd accumulation over a 90-day treatment period. (**d**) Root and (**e**) shoot Cd accumulation after 90 days treatment. (**f**) Total Cd accumulation after 90 days treatment. Different lowercase letters denote significant differences among treatments at the *p* < 0.05 level.

During the 90-day treatment period, the Cd+R+ECO_2_ treatment markedly raised Cd accumulation in roots and total Cd accumulation in *Robinia*–rhizobia symbiosis, while Cd accumulation in shoots showed no significant change (Figure 4a–c). The Cd+R+ECO_2_ treatment significantly increased Cd accumulation in roots (81.2%, *p* < 0.001) and *Robinia*–rhizobia symbiosis (55.5%, *p* < 0.001) compared to the Cd+R treatment (Figure 4d,f). The above findings revealed that ECO_2_ enhanced the Cd accumulation in both the root and *Robinia*–rhizobia symbiosis but showed no evident effect on shoot Cd accumulation.

### 3.5. Effects of ECO_2_ on Cd Chemical Forms Fractions

**Figure 5:**
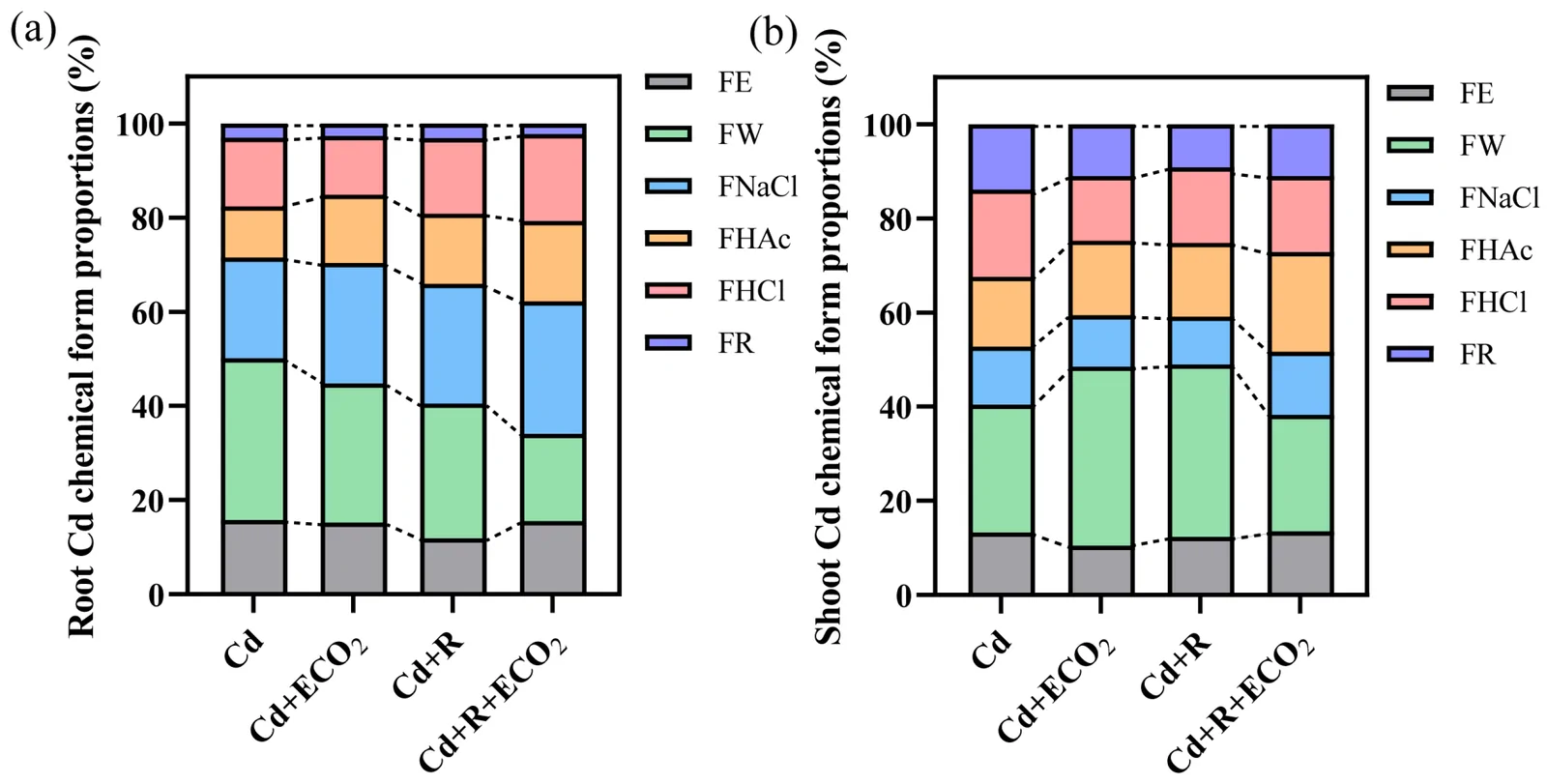
Effects of ECO_2_ on Cd chemical forms fractions. (**a**) Root and (**b**) shoot Cd chemical forms proportions. FE: ethanol-extractable Cd, FW: water-soluble Cd, FNaCl: NaCl-extractable Cd, FHAc: acetic acid-extractable Cd, FHCl: Cd-oxalate, FR: residual Cd.

In terms of Cd chemical forms, the Cd+R+ECO_2_ treatment reduced the proportion of available Cd (FE+FW) by 16.1%, while increasing the proportions of FNaCl, FHAc and FHCl in roots, compared to Cd+R treatment (Figure 5a). Similar results were observed in the shoot of the *Robinia*–rhizobia symbiosis: Cd+R+ECO_2_ treatment lowered the proportion of available Cd (FE+FW) by 21.9% but raised the proportions of FHAc and FR (Figure 5b). The results demonstrated that ECO_2_ altered the chemical forms of Cd in both roots and shoots, thereby modulating Cd bioavailability and toxicity.

### 3.6. Effects of ECO_2_ on Nutrient Elements Content

**Figure 6:**
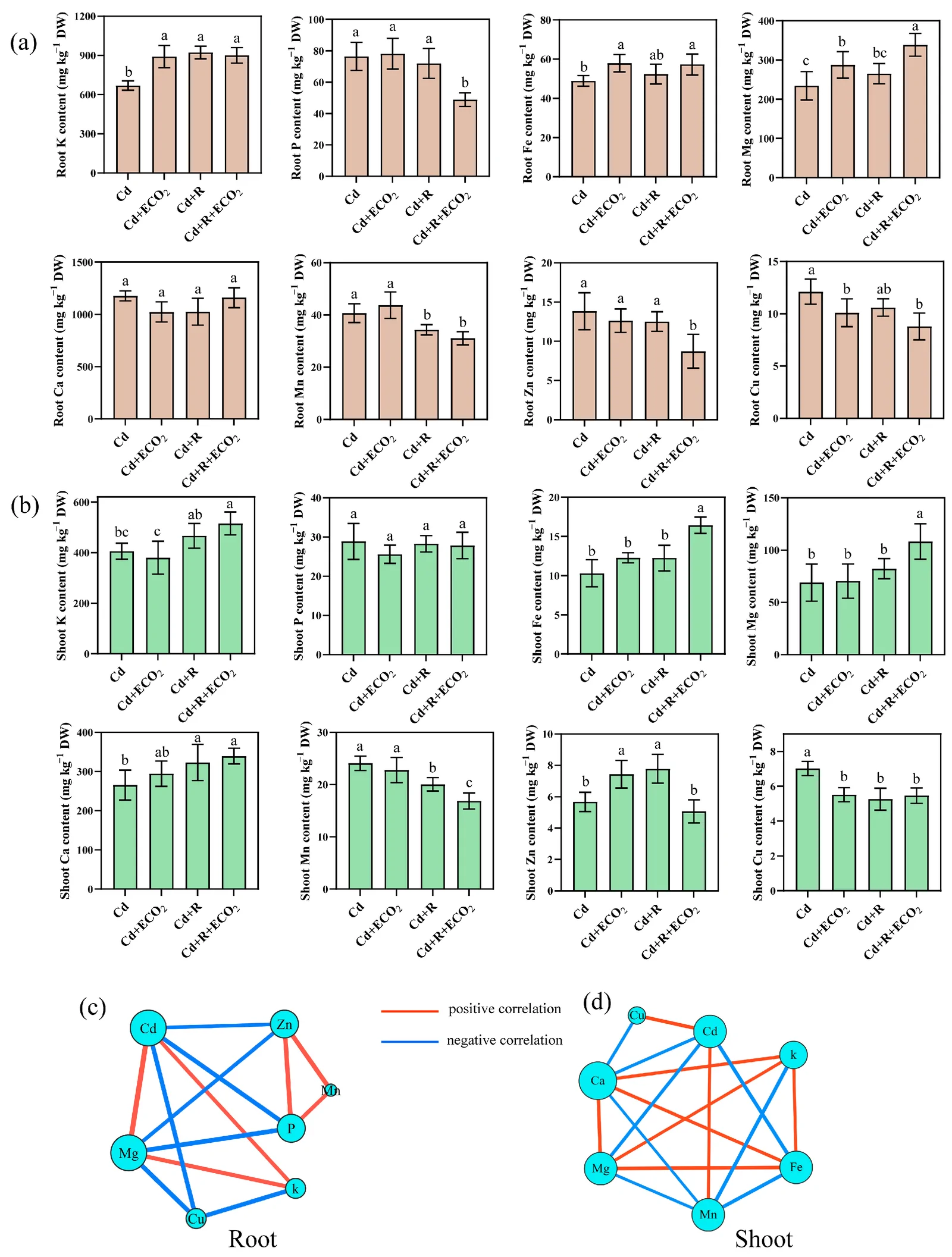
Effects of ECO_2_ on nutrient elements. (**a**) Root and (**b**) shoot nutrient elements content. (**c**) Root and (**d**) shoot correlation network analysis (Pearson’s correlation analysis |r| ≥ 0.5 and *p* < 0.05). The node size is proportional to the number of links. Different lowercase letters denote significant differences among treatments at the *p* < 0.05 level.

ECO_2_ exhibited different effects on the content of nutrient elements in *Robinia*–rhizobia symbiosis. The Cd+R+ECO_2_ treatment significantly increased Mg content in roots by 27.7% but significantly decreased P (32.1%, *p* < 0.01) and Zn (30.3%, *p* < 0.05) content, with no significant effect on other nutrient elements, compared to Cd+R treatment (Figure 6a). In the shoots, Cd+R+ECO_2_ treatment markedly elevated Fe (33.3%, *p* < 0.01) and Mg (31.6%, *p* < 0.05) content while significantly reducing Mn (15.9%, *p* < 0.05) and Zn (34.5%, *p* < 0.01) content, compared to Cd+R treatment (Figure 6b). Correlation network analysis revealed that Cd content showed a significant positive correlation with Mg and K content, and a significant negative correlation with P, Cu, and Zn content in roots (Figure 6c). Additionally, Cd content exhibited a significant positive correlation with Mn and Cu levels, while showing a significant negative correlation with Fe, Ca, and Mg contents in shoot (Figure 6d).

### 3.7. Effects of ECO_2_ on Oxidative Stress and Antioxidant Characteristics in Roots

**Figure 7:**
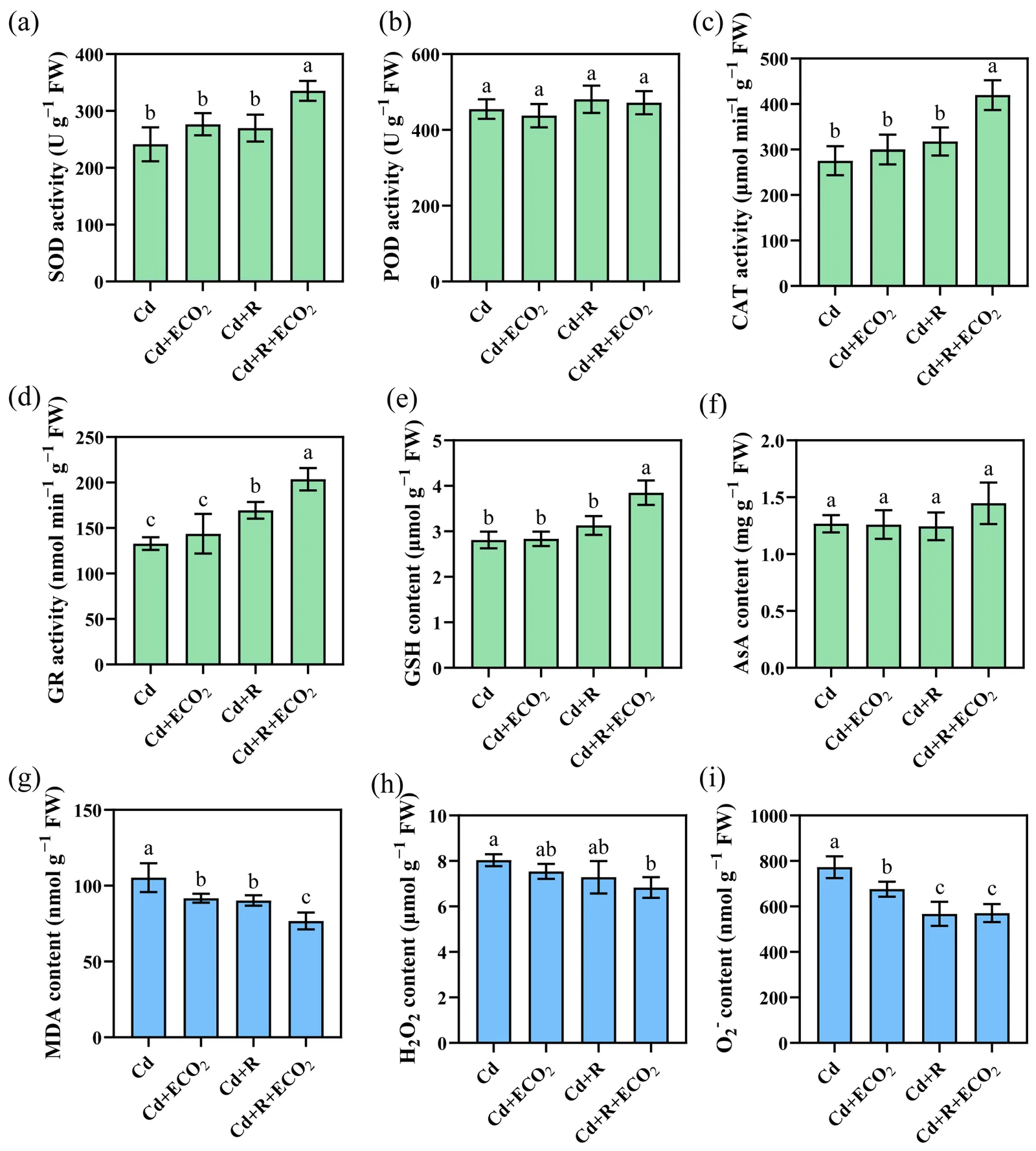
Effects of ECO_2_ on oxidative stress and antioxidant characteristics in roots. (**a**) SOD, (**b**) POD, (**c**) CAT, (**d**) GR, (**e**) GSH, (**f**) AsA, (**g**) MDA, (**h**) H_2_O_2_, (**i**) O_2_^−^. Different lowercase letters denote significant differences among treatments at the *p* < 0.05 level.

The Cd+R+ECO_2_ treatment significantly increased the activities of SOD, CAT, and GR, while also elevating GSH levels, compared to Cd+R treatment. However, it had no obvious effect on POD activity and AsA content in the roots of *Robinia*–rhizobia symbiosis (Figure 7a–f). Moreover, without rhizobia inoculation, ECO_2_ reduced MDA (12.9%, *p* < 0.01) H_2_O_2_ (6.1%, *p* > 0.05) and O_2_^−^ (12.5%, *p* < 0.01) content, compared to Cd exposure alone (Figure 7g–i). Following rhizobia inoculation, ECO_2_ significantly diminished MDA content (14.9%, *p* < 0.01) under Cd exposure (Figure 7g). The results indicated that ECO_2_ alleviated Cd-induced oxidative stress by activating relevant antioxidant enzymes activities and raising antioxidant contents.

### 3.8. Analysis of the Random Forest Model

**Figure 8:**
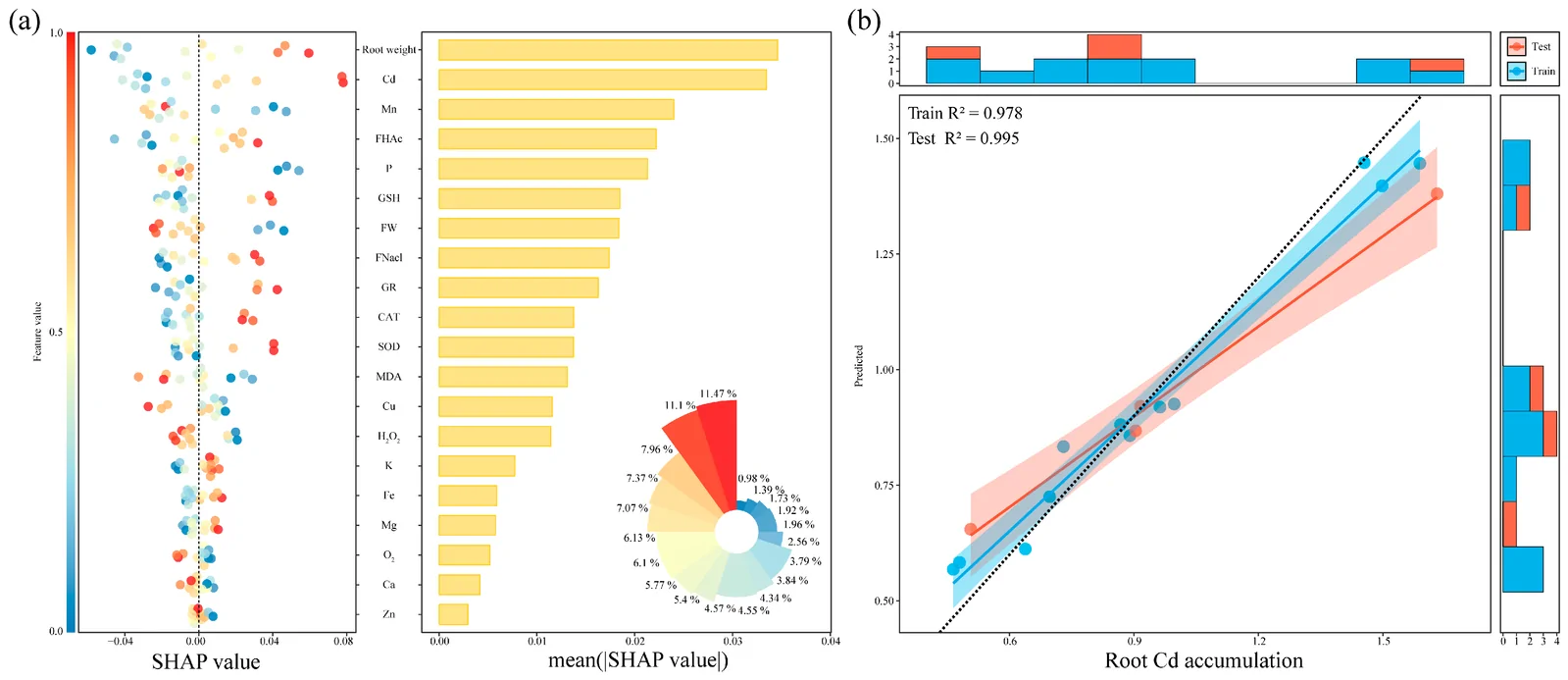
Analysis of the random forest model: (**a**) SHAP analysis of random forest machine learning. The central vertical dashed line indicates a SHAP value of zero; values to the right represent positive contributions to the model prediction, whereas values to the left represent negative contributions. (**b**) Fitting curves of predicted and actual values for the train and test sets.

This study employed a random forest regression to further analyze the influence of physiological and biochemical indicators on Cd accumulation in black locust roots. Shapley additive explanations (SHAP) values were used to define the contribution of each variable to the model prediction. The results indicate that root weight, Cd, Mn, FHAc, P, and GSH exhibit relatively high predictive contributions for root Cd accumulation. Analysis of the SHAP scatter plot revealed that SHAP values for root weight, Cd, and Mn content predominantly cluster in the positive region, indicating that higher root weight and Cd/Mn content tend to affect root Cd accumulation. Furthermore, antioxidant-related indicators GSH, GR, and CAT exhibited positive contributions, suggesting that elevated antioxidant levels correlate with higher root Cd accumulation (Figure 8a). Curve fitting analysis between predicted results and observed values revealed that the model achieved the R^2^ of 0.978 for the training set and 0.995 for the test set, indicating strong fitting capability and predictive accuracy. No significant deviation was observed between model predictions and actual values, demonstrating that the random forest-based prediction model exhibits good stability and reliability (Figure 8b).

## 4. Discussion

The impact of ECO_2_ on plant biomass under Cd exposure is regulated by multiple factors, including plant species, Cd pollution levels, and the rhizosphere microenvironment [35]. A previous study has found that ECO_2_ significantly increased rice yields under Cd exposure from 2019 to 2021 [36]. Elevated ECO_2_ improved the yield of rice grains under Cd and Pb co-contamination [4]. Additionally, ECO_2_ promoted the growth of rapeseed under Cd exposure but did not reach a significant level [34]. This study found similar results that ECO_2_ stimulated the growth of both roots and shoots of *Robinia*–rhizobia symbiosis under Cd exposure (Figure 1). CO_2_ serves as a substrate for plant photosynthesis [37]. Some studies have found that ECO_2_ enhanced net photosynthetic rates in both short-term and long-term treatments [38]. However, other studies revealed that plants acclimated to sustained ECO_2_ conditions, which in turn reduced photosynthesis [39]. ECO_2_ increased the total chlorophyll content in the leaves of *Robinia pseudoacacia* after 45 days, but the chlorophyll content decreased after 90 and 135 days under Cd exposure [17]. In this study, ECO_2_ enhanced photosynthesis and increased the content of photosynthetic pigments after 90 days of treatment under Cd exposure.

There were significant variations in the effects of ECO_2_ on Cd content in different tissues of plants. ECO_2_ reduced the Cd content in rice grains from 2019 to 2021 [36]. However, other studies found the opposite results: ECO_2_ increased the Cd concentration in the roots, stems, and leaves of rice [4]. Moreover, ECO_2_ significantly raised the Cd content in rapeseed roots [34]. ECO_2_ increased Cd content in the leaves of *Robinia pseudoacacia* during the 45 to 135 days treatment period [17]. This study found similar results that ECO_2_ increased Cd levels in the root but reduced Cd levels in the shoot of *Robinia*–rhizobia symbiosis after 90 days of treatment (Figure 3c,d). A previous study revealed that ECO_2_ diminished the transfer factor of Cd, thereby mitigating its accumulation in rice grains [36]. Similarly, in this study, ECO_2_ significantly reduced the TF of Cd, thereby greatly decreasing its concentration in the shoots (Figure 3e). The decomposition of plant litter is one of the primary pathways for heavy metals to return to the soil [40]. Therefore, ECO_2_ minimized secondary environmental pollution caused by Cd released during the decomposition of *Robinia* litter.

Previous studies indicated that ECO_2_ contributed to the accumulation of heavy metals in plants [35]. ECO_2_ significantly raised Cd accumulation in the roots of three rice varieties and in the grains of one variety [41]. ECO_2_ increased Cd concentrations in the grains of rice and wheat, while Cu concentrations exhibited the opposite result [42]. This study found similar findings that within the 90-day treatment period, ECO_2_ consistently increased Cd accumulation in the roots, but no significant differences were observed in the shoots of *Robinia*–rhizobia symbiosis (Figure 4). Taken together, ECO_2_ promoted the accumulation of Cd in the roots, thereby enhancing the phytostabilization of *Robinia*–rhizobia symbiosis on Cd. The different chemical forms of Cd affect its toxicity and bioavailability to plants [43]. In particular, the chemical forms FE and FW exhibit higher mobility and phytotoxicity [44]. FNaCl primarily consists of complexes formed by proteins and pectin with Cd, which are bound to the cell wall. FHAc is insoluble phosphate-Cd, which is sequestered in cell walls and vacuoles, thereby reducing its mobility and bioavailability [31]. FHCl indicates oxalate-Cd, which can be transported into vacuoles to form complexes, greatly reducing its biological toxicity [45]. FR refers to residual-Cd state, which exhibits minimal mobility and the lowest toxicity [46]. In this study, ECO_2_ reduced the proportion of FW in roots and shoots while elevating the proportion of FHAc, indicating that ECO_2_ diminished the bioavailability of Cd and mitigated its toxicity in *Robinia*–rhizobia symbiosis (Figure 5).

As a non-essential heavy metal, Cd interacts with essential plant nutrients in complex ways, typically manifesting as synergistic and antagonistic effects [7]. Cd exposure promoted the content of Ca and Mg in mulberry root [47]. Cd treatment reduced the contents of Fe, Mn, and Zn in rice shoots [48]. Moreover, Cd treatment inhibited the transport of Fe from the roots to the shoots in *Malus* [49]. Competitive absorption of Mn and Cd by plants results in Mn inhibiting the accumulation of Cd in rice [50]. In this study, ECO_2_ increased Mg content, reduced Zn level in the roots, and elevated Fe and Mg content in the shoots. In roots, Cd competed with Cu and Zn but acted synergistically with Mg. Meanwhile, Cd competed with Mg and Fe in shoots (Figure 6). Fe plays a key role in photosynthesis and chlorophyll synthesis, while Mg is an essential element required for the synthesis of photosynthetic pigments [28,51]. The results implied that ECO_2_ potentially enhanced photosynthesis in *Robinia*–rhizobia symbiosis exposed to Cd by increasing Fe and Mg levels in shoots.

The antioxidant system serves a crucial role in protecting plants against Cd stress [52]. Previous studies found that ECO_2_ increased the activities of SOD, POD, and CAT while reducing MDA levels in pepper exposed to Cd [53]. ECO_2_ stimulated SOD activity and lowered MDA content in *Lolium perenne* under Cd stress [54]. Similarly, ECO_2_ elevated SOD activity and diminished MDA levels in poplar and willow, thereby alleviating Cd-induced oxidative stress [3]. ECO_2_ increased the levels of antioxidants, including GSH and ASA, in *Brassica napus* leaves to resist Cd-induced ROS production [34]. In this study, ECO_2_ increased the activities of SOD, CAT, and GR and raised GSH content, thereby mitigating Cd-induced lipid peroxidation in the roots of *Robinia*–rhizobia symbiosis.

Machine learning methods such as the random forest model are increasingly utilized in the remediation and management of heavy metal-contaminated soil [55]. A random forest model based on feature importance indicated that exchangeable Cd, exchangeable Mn, and amorphous Mn were the top three explanatory variables influencing Cd accumulation in plants [56]. The SHAP analysis of the random forest model revealed that the leading three variables contributing to rice As uptake are soil As, soil Cd, and pH [57]. Moreover, the random forest model exhibited high predictive accuracy in evaluating the bioaccessibility of Cd and Pb in soils [55]. Similar findings were observed in this study, with the random forest model identifying root weight, root Cd, root Mn, FHAc, and P content as the top five variables influencing Cd accumulation in the roots of the *Robinia*–rhizobia symbiosis.

## 5. Conclusions

In summary, ECO_2_ facilitated Cd phytostabilization by *Robinia*–rhizobia symbiosis through multiple synergistic mechanisms, including promoting the growth of symbiosis, increasing Cd accumulation, and reducing the Cd bioavailable fraction. The random forest model revealed that root weight, FHAc, and GSH were key factors influencing Cd accumulation in roots. This study offers new insights into the ecological remediation of Cd-contaminated soils using *Robinia*–rhizobia symbiosis in the context of global climate change. The findings of this study are obtained under controlled conditions, which provide theoretical support for subsequent field research, rather than direct in situ remediation effect data. Future work will involve conducting validation experiments using actual contaminated soil samples from the field to verify the regulatory mechanisms identified in this study, thereby facilitating the translation of theoretical findings into practical applications.

## Data Availability

The original contributions presented in this study are included in the article. Further inquiries can be directed to the corresponding author.
